# Emotional, personal, cognitive and other mental disorders after removal of the tumor of the diencephalic region (in the long-term period)

**DOI:** 10.1192/j.eurpsy.2021.1712

**Published:** 2021-08-13

**Authors:** Y. Sidneva, L. Astaf’Eva, O. Zaitsev, P. Kalinin, B. Kadashev, M. Kutin, S. Urakov, I. Voronina, A. Shkarubo, D. Fomichev, D. Andreev, O. Sharipov

**Affiliations:** 1 Psychiatric Research Group, N.N.Burdenko National Medical Research Center of Neurosurgery, Moscow, Russian Federation; 2 Neurosurgery, N.N.Burdenko National Medical Research Center of Neurosurgery, Moscow, Russian Federation; 3 Diagnostic Department, N.N.Burdenko National Medical Research Center of Neurosurgery, Moscow, Russian Federation

**Keywords:** Mental disorders, emotional and personality disorders, craniopharyngioma, postoperative period

## Abstract

**Introduction:**

In the literature, there are conflicting data regarding the recovery of mental disorders, in particular, pathologies of the emotional, personality, behavioral and cognitive spheres, in patients after surgical treatment of tumors of the diencephalic region.

**Objectives:**

To evaluate the dynamics of psychopathological disorders after removal of a craniopharyngioma.

**Methods:**

45 patients (18–68 y.o.), operated through transcranial access. The follow-up period ranged from 3 months to 9 years (on average 2.8 + 0.4). The main method is psychopathological, supplemented by rating scales and questionnaires.

**Results:**

In the late postoperative period, mental disorders were detected in 75% of patients (Table 1). Table 1. Dynamics of the main psychopathological symptom complexes (n = 45).

**Table:** 

Disorders (may be a combination)	Before surgery (n,%)	2 weeks after (n,%)	18 months after (n,%)
Emotional and volitional	27 (60%)	27 (60%)	15 (33%)
Cognitive - Korsakov syndrome	18 (40%) 4 (9%)	27 (59%) 8 (18%)	18 (40%) 7 (15%)
Personality	21 (46%)	25 (55%)	23 (51%)

The table shows that emotional-volitional disorders have a clear positive dynamics by 18 months after surgery compared with the preoperative level. Korsakov’s syndrome and personality disorders are less favorable. 23 patients (52%) returned to their previous profession; 22 (48%) stopped working due to a severe degree of disability, of which 7 (15%) need constant supervision.

**Conclusions:**

The positive dynamics of psychopathological symptoms is observed only within 1.5 years after the removal of the craniopharyngioma, in the future they remain without a tendency to improve. 22 patients (48%) stopped working. The most severe degree of disability is 15% patients.

**Disclosure:**

No significant relationships.

